# The Ovary of *Tubifex tubifex* (Clitellata, Naididae, Tubificinae) Is Composed of One, Huge Germ-Line Cyst that Is Enriched with Cytoskeletal Components

**DOI:** 10.1371/journal.pone.0126173

**Published:** 2015-05-22

**Authors:** Anna Z. Urbisz, Łukasz Chajec, Piotr Świątek

**Affiliations:** Department of Animal Histology and Embryology, University of Silesia, Bankowa 9, 40–007 Katowice, Poland; Institut de Génétique et Développement de Rennes, FRANCE

## Abstract

Recent studies on the ovary organization and oogenesis in Tubificinae have revealed that their ovaries are small polarized structures that are composed of germ cells in subsequent stages of oogenesis that are associated with somatic cells. In syncytial cysts, as a rule, each germ cell is connected to the central cytoplasmic mass, the cytophore, via only one stable intercellular bridge (ring canal). In this paper we present detailed data about the composition of germ-line cysts in *Tubifex tubifex* with special emphasis on the occurrence and distribution of the cytoskeletal elements. Using fixed material and live cell imaging techniques, we found that the entire ovary of *T*. *tubifex* is composed of only one, huge multicellular germ-line cyst, which may contain up to 2,600 cells. Its architecture is broadly similar to the cysts that are found in other clitellate annelids, i.e. a common, anuclear cytoplasmic mass in the center of the cyst and germ cells that are connected to it via intercellular bridges. The cytophore in the *T*. *tubifex* cyst extends along the long axis of the ovary in the form of elongated and branched cytoplasmic strands. Rhodamine-coupled phalloidin staining revealed that the prominent strands of actin filaments occur inside the cytophore. Similar to the cytophore, F-actin strands are branched and they are especially well developed in the middle and outermost parts of the ovary. Microfilaments are also present in the ring canals that connect the germ cells with the cytophore in the narrow end of the ovary. Using TubulinTracker, we found that the microtubules form a prominent network of loosely and evenly distributed tubules inside the cytophore as well as in every germ cell. The well-developed cytoskeletal elements in *T*. *tubifex* ovary seem to ensure the integrity of such a huge germ-line cyst of complex (germ cells - ring canals - cytophore) organization. A comparison between the cysts that are described here and other well-known female germ-line cysts is also made.

## Introduction

The formation of germ-line cysts (clusters, nests, clones) seems to be a conserved phase of gametogenesis in most invertebrate and vertebrate animals [1–3]. Usually during early oogenesis, germ-line stem cells (GSCs) divide asymmetrically and produce new GSCs and cyst progenitor cells (cystoblasts, Cbs). Then the Cbs divide mitotically several times without full cytokinesis, and as a result, sibling germ cells (cystocytes) are interconnected by broad cytoplasmic channels (stable intercellular bridges, ring canals) and form syncytia [1, 2, 4]. When a given cyst is completed there are two modes for its future development—panoistic (e.g., some vertebrates such as X*enopus laevis* and M*us musculus*) in which cytokinesis is completed quickly and each germ cell has the potential to become an oocyte, or meroistic (e.g., some insects such as *Drosophila melanogaster*) in which germ cells are interconnected for a longer period of time and their fates diverge, i.e. usually only a small number (sometimes one) of the germ cells differentiate into oocytes while the rest of the cells play the role of supporting cells (nurse cells, trophocytes) and finally die [1–8]. It should be added here that in some animals, e.g. in basal insects, female germ-line cysts have never been found [5] and recent studies of the firebrat *Thermobia domestica* have confirmed that individual Cbs develop directly into oocytes (panoistic oogenesis) in this species [9].

The female germ-line cysts in different animal taxa show a great deal of diversity. The main differences are the number of interconnected cells and the spatial pattern of cell distribution – the cyst architecture. The simplest, two-celled cysts have been found in some insects such as *Forficula auricularia* [10] and in the polychaete annelid *Ophryotrocha labronica* (Table 1) [11, 12], 16-celled cysts are known from the ovaries of *Drosophila melanogaster* [8] and *Xenopus laevis* [13], whereas as many as 250 cells have been found in the female cyst in the Strepsiptera [14, 15].

**Table 1:** The summary of germ-line cyst organization in different groups of animals in conjunction with the cytoskeleton.

Species	Cyst geometry	Number of germ cells in a cyst	Interconnection of cysts components	Molecular composition of intercellular bridges	Actin cytoskeleton	Microtubular cytoskeleton
***Tubifex tubifex***	Cone-like, evidently polarized; long and branched cytophore ([27] this study)	~2000 (including ~ 8 growing oocytes) [this study]	Each germ cell has one ring canal connecting it to the cytophore ([27] this study)	F-actin [this study]	Subcortical actin bundles in all germ cells; extensive, long and branched actin bundles within the cytophore reaching intercellular bridge of each germ cell [this study]	Prominent network of microtubules in cytoplasm and around nuclei of nurse cells and oocytes; network of microtubules and parallel bundles within the cytophore [this study]
***Drosophila melanogaster***	During formation rosette-like with maximally branched pattern of cell interconnections, then flat or lens-shaped [8, 56]	16 (15 nurse cells and 1 oocyte) [4, 8, 56]	Two germ cells with 4 ring canals, 2 with 3, 4 with 2, and 8 cystocytes with 1 ring canal [8, 56]	completely assembled: F-actin, Ov-htsRC, Kelch, Cheerio, Cullin-3, Pav-Klp,Tec29, Src64B, Mucin-D, pTyr proteins, Visgun; temporarily: Cindr, Anilin [4, 56]	Subcortical and cytoplasmic F-actin; baskets of F-actin bundles at the ring canals on the nurse cells side during selective transport; the cage of radially arranged F-actin around the nurse cells nuclei to prevent entering their nuclei into the oocyte [56]	Microtubular anterior-posterior gradient of diminishing abundance and polarization within oocyte causing proper localization of macromolecules and the nucleus; subcortical parallel arrays of microtubules in the oocyte which mix nurse cell cytoplasm entering to the oocyte during the rapid transfer [54, 55, 63]
***Xenopus laevis***	Rosette-like with maximally branched pattern of cell interconnections [13]	16 (all cells develop as oocytes) [13]	Two germ cells with 4 ring canals, 2 with 3, 4 with 2 and 8 cystocytes with 1 ring canal [13]	F-actin, Kelch, Ov-htsRC [13]	Rich subcortical and radially spread actin-based cytoskeleton in fully grown oocytes [13, 64]	Polarized microtubules involved in transport of synchronizing factors in early germ cells [13, 64]
***Ophryotrocha labronica***	Two-celled cysts forming by fragmentation of parental cysts with no consistent pattern of branching (linear or more complex) [12]	2 (1 nurse cell and 1 oocyte) [11, 12]	One ring canal connecting nurse cell with oocyte [11, 12]	pTyr proteins [11]	Actin filaments localized in the nurse cell cortex causing a contraction of this cell and allow transferring cytoplasm into the oocyte [11]	Extensive microtubule cytoskeleton in the ring canal of developing cysts forming a scaffold which prevent inappropriate mixing of cytoplasm [11]
***Caenorhabditis elegans***	U-shaped and polarized with syncytial part (mitotic germ cells) and cellularized growing oocytes; long unbranched cytoplasmic core (rachis) extend through the syncytial part of cyst and disappears within the part of developing oocytes [22, 23, 43, 44]	~1300 syncytial germ cells and 10–14 oocytes (at a time) [22]	Each germ cells possess one intercellular bridge connecting it to the rachis [22, 23, 43, 44]	Anilin protein ANI-2 [45, 46]	Randomly oriented filaments within the rachis in the pachytene region of the gonad; rich actin filaments around oocytes nuclei; fine filaments within the rachis and numerous within ring canals of enlarging oocytes in the direction of cytoplasmic streaming [47]	Baskets of microtubules surround germ cells nuclei from the pachytene region throughout subsequent stages of oogenesis until the oocyte cellularization. Microtubules extend through intercellular bridges and within the rachis [47]
***Stomaphis quercus***	During formation each ovariole consists of single spherical cyst with germ cells arranged into a rosette; then cyst elongates and forms a centre occupied by more or less long, common cytoplasmic area (trophic core); the anterior part of cyst (tropharium) contains nurse cells; posterior part (vitellarium) contains growing oocytes [48]	32 germ cells in viviparous generations (including 1–2 oocytes); 45–60 in oviparous (including 1 oocyte) [48]	Germ cells connected with trophic core by cytoplasmic bridges; Nurse cells form processes which extend into the trophic core; Vitellogenic oocytes are connected to the trophic core by the broad nutritive cords (modified intercellular bridges) [48]	–	–	Bundles of microtubules filling the trophic core; Parallel-arranged microtubules within nutritive cord [48]

No available data (-).

As for the architecture of cysts, the cells in a cyst may form linear/almost linear chains – all of the cystocytes except the terminal ones are connected to their sister cells by two ring canals; the terminal cystocytes only have one ring canal [8]. Such linearly organized cysts have been found, e.g., in the polychaete annelid *Diopatra cuprea* [16] and in some insects such as collembolans and mayflies [5, 17]. A more complicated pattern of cell distribution is observed in branched cysts, i.e. where the cystocytes are connected to their neighbours via more than two intercellular bridges and form “branches” [4, 5, 6, 8, 13, 18–20]. Branched cysts have been found and described in detail in model organisms such as *Drosophila melanogaster* and *Xenopus laevis* (see Table 1 for some details and references).

The organization of the female germ-line cysts in such taxa as clitellate annelids, nematodes and mites is more complicated; the center of the cyst is occupied by an anuclear cytoplasmic mass (cytoplasmic core, central core) of a different shape and size, which is called a cytophore in clitellate annelids, a gonad core or a rachis in nematodes and a medulla in mites, while the germ cells are located at the periphery of the cyst. Additionally, as a rule, each germ cell is connected to the cytoplasmic core via only one stable intercellular bridge [21–32]. The number of germ cells in cysts that have a central core is usually high (the lowest known number is 16 in the white worm, *Enchytraeus albidus* [33]) and usually varies between taxa and may even vary between different germ-cell clusters in the same ovary (e.g., there are from 24 to 44 cells in fish leeches, *Piscicola* [34]). There are about 1,300 female germ cells that are connected to a long, tube-like central core in the gonads of the nematode *Caenorhabditis elegans* (Table 1) [22].

Recently, the gross morphology of the female germ-cell cysts in some clitellate annelids has been described in several taxa e.g., in leeches [26, 28], and in oligochaetous clitellates [27, 31]. These studies revealed that there is a great deal of variety in the cyst morphology in these animals (for more details see Discussion, [32] and references herein). One of the groups of Clitellata in which germ-line clusters have drawn special attention due to their complicated form is Tubificinae. The morphology of the ovary and the course of oogenesis in three representatives of this group have recently been studied [27]. However, there is still a lack of answers to basic questions such as how many germ-line cysts occur in the entire tubificinin ovary and how many germ cells constitute a single cyst? What is the exact cyst architecture? What is the organization of the microtubular and F-actin cytoskeleton? In the paper presented here, we focus on these details of the germ-line cysts in the sludge worm *Tubifex tubifex* (Tubificinae). In order to address these questions, we analyzed both sectioned and whole-mounted *T*. *tubifex* ovaries using a chemically fixed material and live cell imaging techniques. We found that the entire ovary is composed of only one, huge multicellular (~2,000 cells) germ-line cyst that is enriched with cytoskeletal elements.

## Materials and Methods

### Animal material

Specimens of *Tubifex tubifex* (Müller, 1774) were purchased from commercial sources and maintained in a 10,000 cm^3^ aquarium under laboratory conditions. Only mature specimens with a fully developed clitellum were selected for the study. About 100 specimens were used for all of the analyses.

### Differential interference contrast

The specimens of *T*. *tubifex* were fixed in 4% formaldehyde (freshly prepared from paraformaldehyde) in PBS (phosphate buffered saline, NaCl, 137 mM; KCl, 2.7 mM; Na2HPO4 8 mM; KH2PO4, 1.5 mM, pH 7.4) for 30–40 min at room temperature and washed in PBS. After that the ovaries were dissected, they were whole-mounted onto microscope slides, and observed under an Olympus BX60 microscope equipped with Nomarski differential interference contrast.

### Light microscopy

The individuals of *T*. *tubifex* were initially fixed with 2.5% glutaraldehyde in a 0.1 M phosphate buffer (pH 7.4), then isolated segments with gonads were fixed in 2.5% glutaraldehyde in the same buffer at room temperature for several days. After washing in a phosphate buffer, the material was postfixed for 2 h in 1% OsO_4_ in the same buffer, dehydrated in a graded series of ethanol that was replaced by acetone and embedded in Epon 812 (Fullam Inc., Latham, NY, USA). Semi-thin sections (0.8 μm thick) were stained with methylene blue and examined under an Olympus BX60 microscope equipped with a DP12 digital camera and AnaliSIS 3.2 (Soft Imaging System) software.

### DAPI and propidium iodide staining

After initial fixation in 4% formaldehyde (freshly prepared from paraformaldehyde) in PBS, the individuals of *T*. *tubifex* were dissected and the ovaries that were obtained were fixed in 4% formaldehyde (for 40 min at room temperature), washed in PBS, dehydrated in a graded ethanol series, infiltrated and embedded in Histocryl resin (London Resin Company Ltd, Basingstoke, Hampshire, England). The Histocryl sections (1 μm) were double stained with DAPI (1 μg/ml) and propidium iodide (1 μg/ml; Sigma) at a ratio 1:1 for 30 min in darkness. Sections were observed under an Olympus BX60 epifluorescence microscope equipped with the appropriate filters.

### Detection of F-actin

Dissected gonads of *T*. *tubifex* were fixed in 4% formaldehyde (freshly prepared from paraformaldehyde) in PBS for 30–40 min at room temperature and stained with rhodamine-conjugated phalloidin (2 μg/ml, Sigma) for 40 min in darkness, washed again in PBS and additionally stained with DAPI (1 μg/ml) for 30 min in darkness. Stained ovaries were whole-mounted onto microscope slides and analyzed under both an Olympus BX60 epifluorescence microscope equipped with appropriate filters and an Olympus FV1000 confocal microscope.

### Detection of Microtubules

Sludge worms were anesthetized in ethanol, then dissected in DPBS (Dulbecco’s Phosphate Buffered Saline, Sigma-Aldrich D4031) and incubated in a prewarmed staining solution of Tubulin Tracker Green (Molecular Probes, Eugene, OR, USA) for 30 min at 37°C (the Tubulin Tracker solution was prepared according to manufacturer’s protocols). In addition Hoechst 33342 (1 μg/ml, Molecular Probes) was added to the staining solution, in order to counterstain the nuclei. After rinsing with DPBS, the ovaries were whole-mounted onto microscope slides and analyzed under an Olympus BX60 epifluorescence microscope.

### Counting the germ cells in the ovary

To count the germ cells, the specimens that were initially fixed in 4% formaldehyde (freshly prepared from paraformaldehyde) in PBS were dissected and the ovaries that were obtained were additionally fixed in the same fixative for 30–40 min at room temperature and stained with DAPI for 30 min in darkness. The ovaries were then whole-mounted onto microscope slides and analyzed under an Olympus FV1000 confocal microscope. The nuclei of the germ cells were counted using ImageJ Software. Ten ovaries were analyzed for the cell count.

## Results

General organization of the germ-line cyst in conjunction with the actin filaments

**Fig 1:**
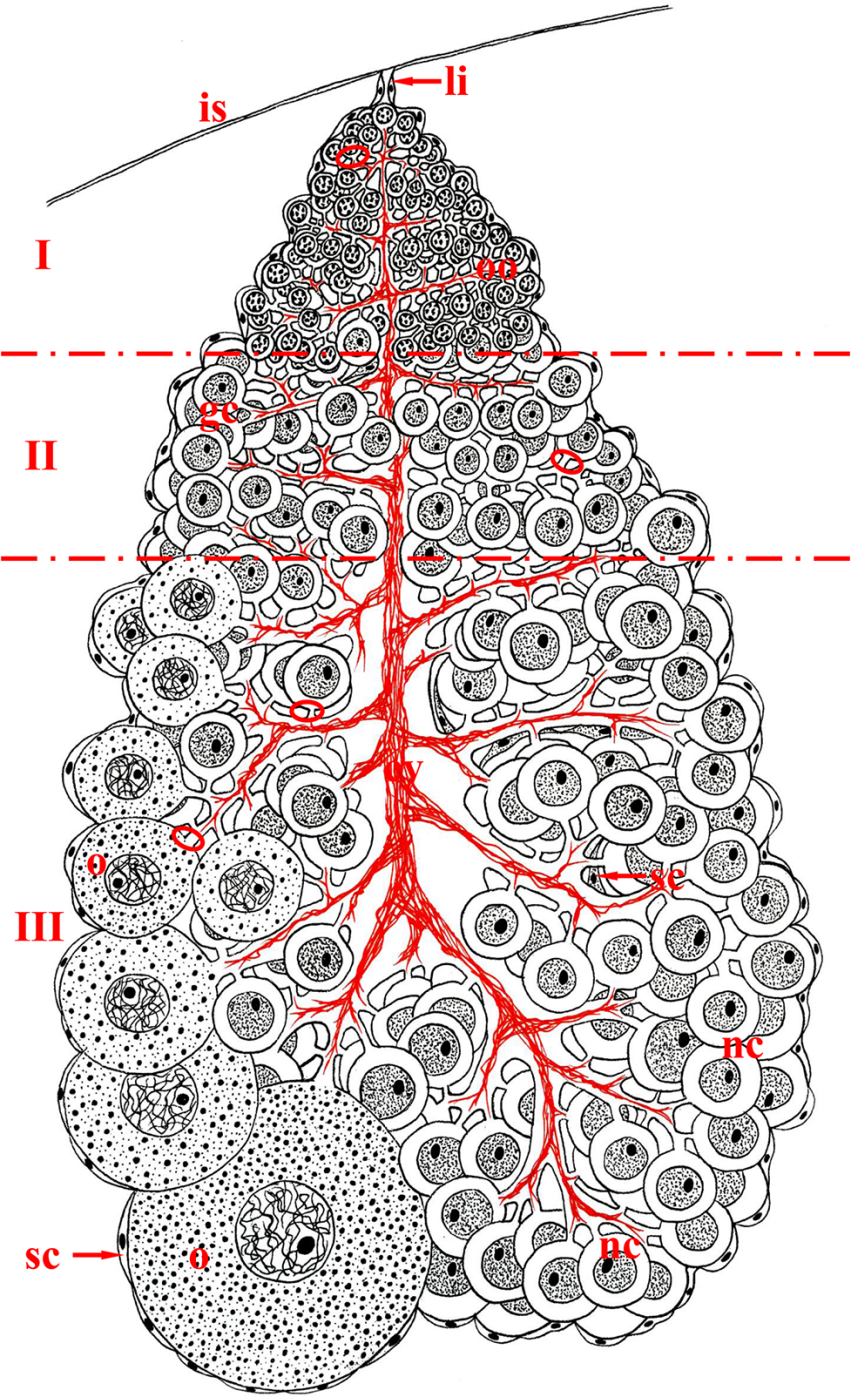
Schematic illustration of the spatial organization of the *T*. *tubifex* ovary. The ovary is conically shaped with its narrow end attached to the intersegmental septum (is) via a thin ligament (li) and is polarized along its long axis. Germ cells at subsequent stages of oogenesis occur in three zones (I, II, III): zone I contains oogonia (oo), undifferentiated germ cells (cystocytes) (gc) occur in zone II and germ cells that are morphologically differentiated into growing oocytes (o) and smaller nurse cells (nc) occur in zone III. Oocytes grow on only one side of the ovary and gradually protrude into the body cavity. The entire ovary is composed of only one, huge germ-line cyst. Each germ cell in a cyst (ovary) is connected to the central anuclear core (cytophore) via one stable intercellular bridge (ring canal – ellipse). The cytophore (cy) is poorly developed in zones I and II, whereas it is prominent and branched in zone III. In addition to the germ cells, somatic cells (sc) occur – they are localized inside the ovary and on its surface and form a thin ovary envelope.

**Fig 2:**
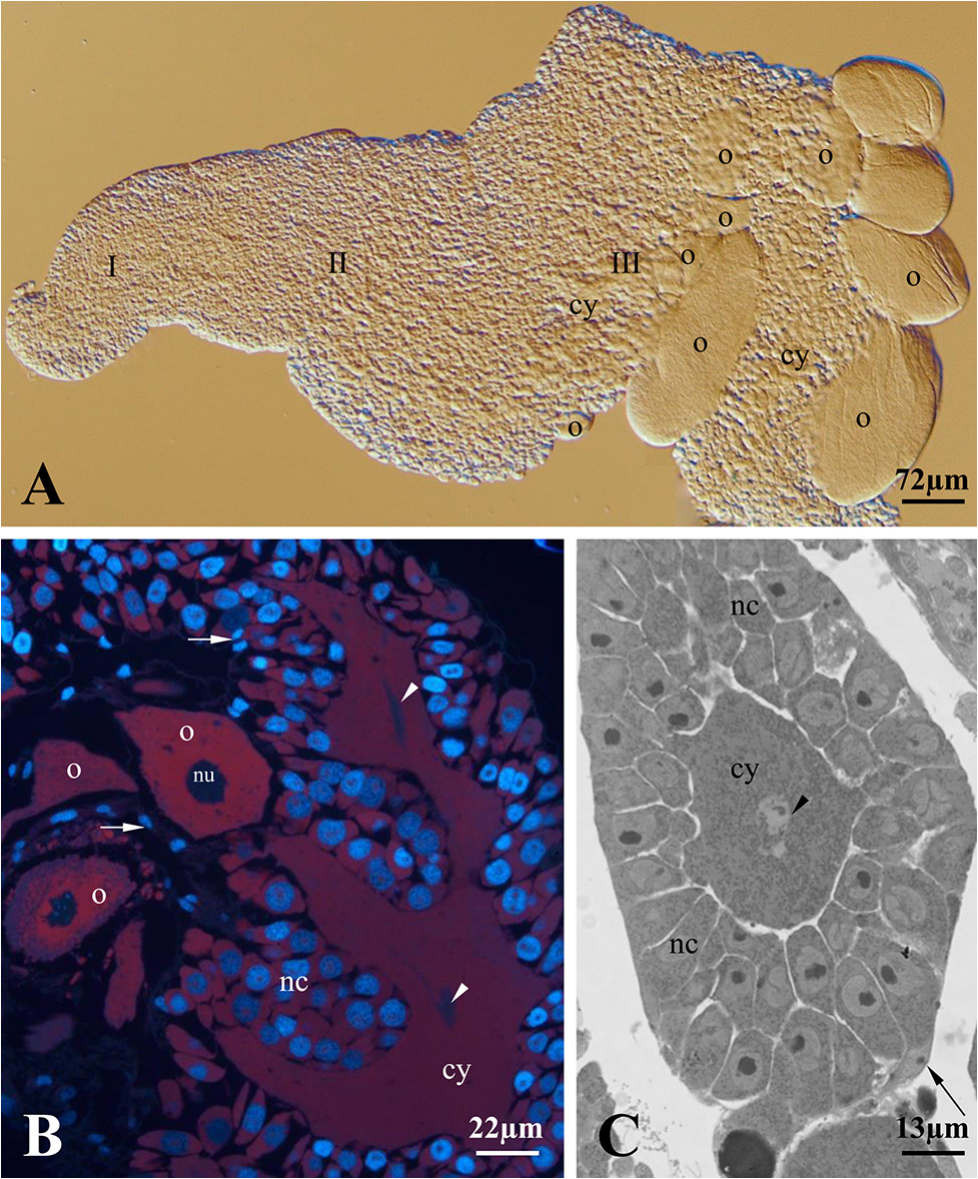
General organization of an ovarian cyst in *T*. *tubifex*. (A) The ovary of *Tubifex tubifex* is conically shaped and polarized. Three zones can be distinguished (I, II and III). Each zone contains germ cells at subsequent stages of oogenesis: oogonia in zone I, undifferentiated cells in zone II and nurse cells and oocytes in zone III. In the third zone, several oocytes (o) grow successively on only one side of the ovary and protrude into the body cavity. An extensive cytophore (cy) is marked in this zone. Whole-mounted preparation, Nomarski interference contrast. (B) and (C) sections through zone III, in which two morphologically distinct germ cell categories occur – nurse cells (nc) and oocytes (o). The branched cytophore (cy) occupies the central position in the germ-line cyst. Arrowheads mark the cytophore regions that are enriched with cytoskeletal elements (see Figs 2A and 3). Arrows point to the somatic cells that accompany the germ cells inside the ovary and form the outer ovary envelope; nu – oocyte nucleus. B longitudinal section, fluorescence microscopy, Histocryl semi-thin section double stained with DAPI and PI; C cross section, light microscopy, Epon semi-thin section stained with methylene blue.

**Fig 3:**
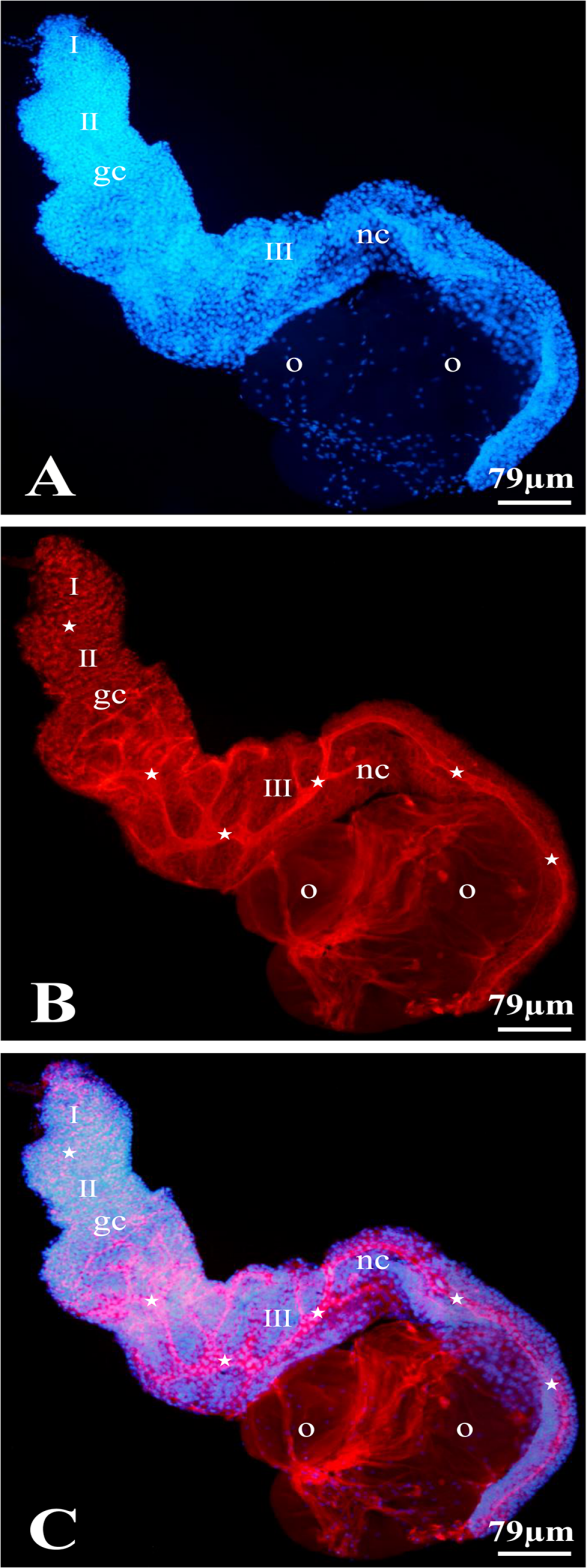
The cytophore organization in an ovarian cyst of *T*. *tubifex*. The entire ovary was stained with DAPI for the visualization of nuclei (A) and rhodamine-conjugated phalloidin to detect F-actin (B); A merged view (C). Maximum-intensity projections of the Z stacks that cover the entire ovary indicate that the entire ovary is composed of one multicellular germ-line cyst. A branched cytophore (stars) extends along the long axis of the ovary in the cyst; the cytophore is thin and inconspicuous in zones I and II, while it is more voluminous and branched in zone III; gc – germ cells, o – oocytes, nc – nurse cells. Confocal microscopy.

**Fig 4:**
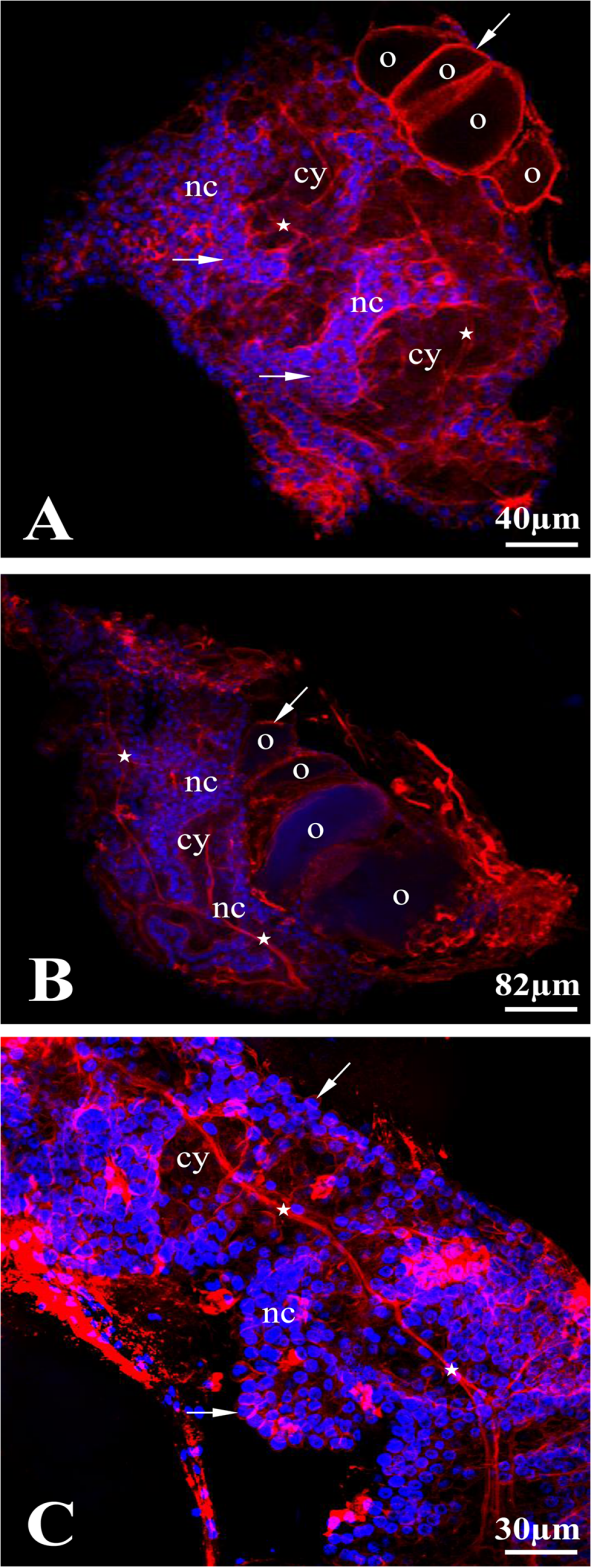
F-actin localization in a *T*. *tubifex* ovarian cyst. The entire ovary double stained with DAPI and rhodamine-conjugated phalloidin. Maximum-intensity projections of the Z stacks that cover the interior of the ovary. (A and B) whole ovaries, (C) detail of area between zones II and III. Long bundles of actin filaments occur (stars) in the branched cytophore (cy). F-actin is also localized in the cortical cytoplasm (arrows) of nurse cells (nc) and oocytes (o). Confocal microscopy.

A detailed description of *T*. *tubifex* ovaries was presented in our earlier paper [27]. The schematic illustration of *T*. *tubifex* ovary organization is presented in Fig 1. Briefly, the ovary of *T*. *tubifex* is a small (~2mm long), conically shaped and clearly polarized structure with its narrow end equipped with a thin ligament that connects it to the intersegmental septum (Figs 1, 2A and 3). The gradient of germ cell development is observed along the long ovary axis, i.e. the oogonia occur just below the ligament – zone I, below that there are undifferentiated germ cells (cystocytes) – zone II, and germ cells that have two morphologically distinct categories occupy the rest of the ovary – zone III (Figs 1–4). We regard these two morphologically different types of germ cells as nurse cells and oocytes (for ultrastructural details and discussion about possible ovary meroism in *T*. *Tubifex* see [27]). Growing oocytes, as a rule, are situated on the surface of the gonad and are arranged on only one side of the ovary (Figs 1, 2, 3, 4A and 4B). Somatic cells accompany germ cells both inside the ovary and around the entire ovary and form a thin, outer envelope.

**Fig 5:**
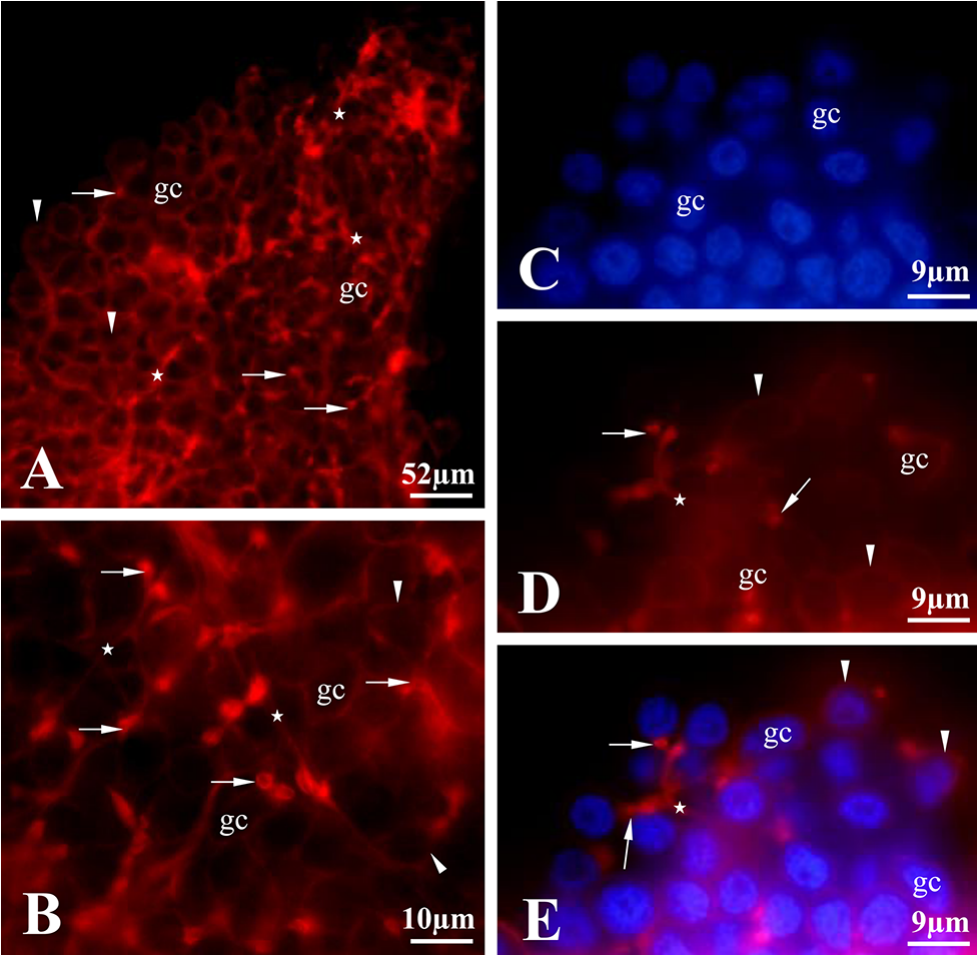
Organization of F-actin in an ovarian cyst of *T*. *tubifex*. (A and B) The initial cytophore with thin F-actin bundles occurs at the narrow end of the ovary (zones I and II) (stars). The cytophore with its F-actin bundles reaches every germ cell (gc). The ring canals that connect the germ cells to the cytophore have rims that are enriched with F-actin (arrows). The F-actin also occurs in the cortical cytoplasm of the germ cells (arrowheads). Fluorescence microscopy, whole-mounted preparation stained with rhodamine conjugated phalloidin. C-E Fragment of zone II double stained with DAPI (C) and rhodamine-phalloidin (D); merged view (E). The cytophore has thin bundles of F-actin (stars) that reach each germ cell (gc). The ring canals rims are enriched with F-actin (arrows) and cortical F-actin can be observed in the germ cells (arrowheads). Fluorescence microscopy.

**Fig 6:**
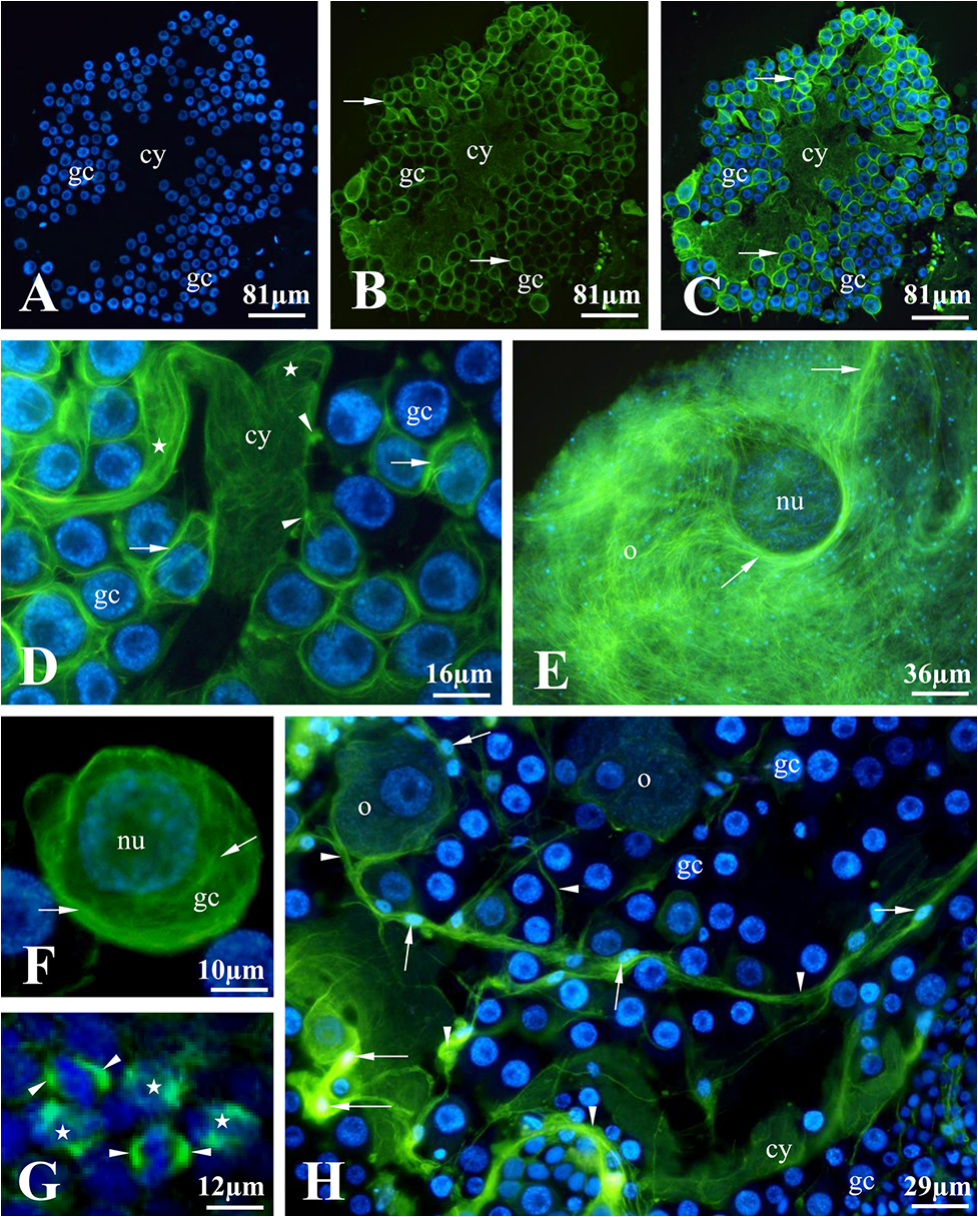
Localization of microtubules in a *T*. *tubifex* ovarian cyst. Zone III. Live cells stained with Hoechst 33342 (A) and Tubulin Tracker Green (B); (C-H) merged. (A-D) Microtubules form a network that fills the entire cytoplasm of the cytophore (cy); note microtubules in the ring canals that connect the germ cells (gc) with the cytophore (arrowheads) and the bundles of microtubules in the cytophore branches (stars in D). (E-F) A prominent network of microtubules (arrows) occurs in the cytoplasm of the germ cells (gc) and in the vitellogenic oocytes (o); nu – nucleus. (G) Mitotic spindles (arrowheads) of dividing germ cells were observed in zone III; stars indicate metaphase plates. (H) Long bundles of microtubules (arrowheads) were also detected in the cytoplasmic projections of the somatic cells (arrows point to the nuclei of these cells); gc – germ cells, o – oocytes. Fluorescence microscopy.

Analysis of double stained (rhodamine-phalloidin + DAPI) ovaries clearly showed that the entire *T*. *tubifex* ovary is composed of only one huge germ-line cyst (Figs 3 and 4). This conclusion is supported by one major observation – there is only one common cytoplasmic mass (the cytophore), which snakes back and forth around the long ovary axis, in each ovary that was studied. However, the cytophore forms numerous more or less broad branches, which are clearly connected to the main cytophore strand and no borders can be detected between cytophore strands (Figs 3 and 4). Rhodamine-conjugated phalloidin staining revealed numerous actin filaments within the cytophore (Figs 3–5). In zones I and II, where the cytophore has the form of very thin, inconspicuous strands of cytoplasm (initial cytophore; for ultrastructural details see [27]), the bundles of actin filaments are thin (Figs 3, 5A and 5B). In zone III, where the cytophore becomes more voluminous and occupies the central position in the cyst in the form of a long, broad and branched cytoplasmic core, an actin filaments form prominent cables (Figs 2B and 2C – 4). Actin cables are even visible as lighter areas inside the cytophore cytoplasm in the semi-thin sections (Fig 2B and 2C). In zone III the cytophore forms more and more finer branches, in which very thin F-actin bundles occur, and they seem to reach individual germ cells (Figs 2B and 2C, 5). All of the germ cells in the ovary are connected to the cytophore (Fig 5) [27]. The cell count revealed that the total number of germ cells in a cyst (ovary) varies from 1,442 to 2,649 (Table 2); the average number of cells per cyst is 2,040 (N = 10 ovaries). Four to 18 oocytes (on average 8) grow and protrude on the ovary surface (Table 2, Figs 2A, 3, 4A and 4B). The number of germ cells in a given cyst is not constant, because mitotic divisions of germ cells have been found even in fully developed ovaries (Fig 6G).

**Table 2:** 

Ovary	Number of germ cells	Number of growing oocytes
1	1905	8
2	1469	0
3	1442	8
4	1752	9
5	2521	13
6	1845	18
7	2400	11
8	2002	5
9	2413	7
10	2649	5
Average	2039,8	8,4

Each germ cell united into a syncytial germ-line cyst has only one ring canal that connects it to the cytophore (Fig 5B–5E) [27]. Recent ultrastructural studies revealed that the walls of ring canals have a layer of fibrous electron-dense material [27]; the present studies showed that they are enriched with actin filaments (Fig 5). The F-actin was easily detectable in the ring canal walls of oogonia and undifferentiated germ cells (zones I and II), while the F-actin labeling was much weaker in the ring canals of the nurse cells and oocytes in zone III (Figs 3–5). Actin filaments are also distributed in the cortical cytoplasm in both individual germ cells and the cytophore (Figs 4, 5A, 5B, 5D and 5E).

### Localization of microtubules

We found numerous microtubules in the germ-line cyst in *T*. *tubifex* ovaries using Tubulin Tracker Green. The bundles of microtubules are distributed in both the cytoplasm of the germ cells and in the cytophore (Fig 6A–6D and 6F). Microtubules form a dense network that fills the entire cytoplasm and encircles the nuclei in the germ cells (Fig 6A–6D and 6F). Similarly, a prominent network of microtubules was also seen in the cytoplasm of vitellogenic oocytes, which lose contact with the cytophore and float freely in the coelomic fluid (Fig 6E). Microtubules were observed filling the whole volume of the cytophore, and they even extended out into its smaller branches (Fig 6A-6D). Microtubules were also detected inside the ring canals that connect the germ cells with the cytophore (Fig 6D).

As in other clitellate annelids, the germ cells in the *T*. *tubifex* ovary are associated with somatic cells (Fig 1, 2B, 2C and 6H) [27]. Somatic cells are localized both inside the germ-line cyst – between the germ cells and the cytophore and on the surface of the cyst/ovary where they form a thin envelope. The somatic cells are easily distinguishable from the germ cells; they are always smaller than the germ cells, and extremely elongated with long cytoplasmic projections. The nuclei of the somatic cells are small and flattened with condensed chromatin, which gives a strong signal after staining with DAPI or Hoechst (Figs 1, 2B, 2C and 6H). In the cytoplasm of somatic cells the microtubules were also observed, especially the bundles of microtubules are localized along the long cytoplasmatic projections of these cells (Fig 6H).

## Discussion

The entire ovary of *T*. *tubifex* is composed of only one germ-line cyst that has an architecture that is similar to other clitellate annelids

The present study allowed us to analyze the ovary organization in *T*. *tubifex*. We found that only one multicellular germ-line cyst constitutes the entire ovary of this species and that this cyst has one long, branched cytophore that extends along the long ovary axis. The *T*. *tubifex* ovarian cyst is polarized, and therefore the process of oogenesis occurs along this axis. The number of germ cells in a single cyst (= ovary) is nearly 2,000 and on average eight oocytes grow at the same time (see Table 2). However, the cell number in the cysts is not constant due to the mitotic divisions of the germ cells, which may even occur in fully developed ovaries that have growing oocytes. A similar asynchrony in germ cell divisions has been observed in some other clitellate groups i.e. [26, 30, 35, 36], which indicates that germ cells can still proliferate even in ovaries in which vitellogenic oocytes are already formed.

Studies of ovary organization and oogenesis in clitellate annelids have shown that the formation of germ-line cysts is a conserved phase of gametogenesis. In all of the clitellate annelids that have been studied to date, as a rule, each germ cell is connected to the common cytoplasmic mass (cytophore) by only one stable cytoplasmic bridge [24–28, 30, 31, 33–38]. Despite that general rule, the cyst morphology varies substantially between different clitellate taxa with respect to the number of germ cells, the level of cytophore development and the ratio of nurse cells to oocytes. In the majority of clitellate annelids (Tubificinae, Lumbricida, Lumbriculida, Branchiobdellida, Acanthobdellida, Glossiphoniidae, Erpobdelliformes and Hirudiniformes) ovaries are composed of irregularly shaped and multicellular cysts, however, the exact number of cysts in an ovary and germ-cells in a cyst has not yet been determined [24–26, 28, 31, 35, 37, 38]. In contrast, spherical cysts that are composed of a low number of cells occur only in the whiteworm *Enchytraeus albidus* (Enchytraeidae) and in fish leeches (Piscicolidae) (see Table 2) ([33, 34, 39], our unpublished results).

Recent papers that have been devoted to oogenesis in clitellates annelids describe two categories of germ cells, which differentiate during the process – several cells (rarely one) continue oogenesis, grow considerably and become oocytes, while the majority of germ cells in a given cyst withdraw from meiosis, do not gather nutrients or grow, and do not seem to develop into oocytes (for details see Table 2). Due to these morphological differences, ovary meroism is suggested in clitellate annelids and these two categories of germ cells are usually regarded as oocytes and nurse cells, respectively [25–28, 30, 31, 34–38]. In this paper, we also regard the two morphologically distinct cell categories that are found in zone III as nurse cells and oocytes. However, in order to determine whether the ovaries in Clitellata are really meroistic (i.e. the nurse cells supply growing oocytes with macromolecules and cell organelles and eventually die) specifically dedicated studies are needed. To date, the only experimental evidence that supports ovary meroism in Clitellata comes from old autoradiographic studies of oogenesis in the leech *Glossiphonia complanata*. These studies showed that because H3-uridine is actively incorporated into the nucleus and nucleolus of nurse cells, and then labeled RNA is passed into the growing oocytes [40].

### Female germ-line cysts in *T*. *tubifex* in comparison with other species

Female germ-line cysts have been studied intensively in only a few groups of invertebrates and vertebrates, primarily in the fruit fly, *Drosophila melanogaster*. Studies of this species provided interesting data, including the molecular and cellular aspects of germ-line cyst development and function, i.e.: the role of germ-line stem cells, the structure of ring canals, the formation and function of the fusome, germ-line cyst polarization and oocyte determination and the organization of the cytoskeleton (Table 1); for details see [3–5, 7, 8, 41, 42].

Although female germ-line cysts in the fruit fly are the best known, they are only one specific example of female cyst organization. To show the diversity of female cyst organization in animals and for comparative purposes, we chose several others that have been studied in this aspect, species such as the polychaetous annelid *Ophryotrocha labronica* [11, 12], the nematode *Caenorhabditis elegans* [22, 23, 43–47], the aphid *Stomaphis quercus* [48] and among vertebrates the frog *Xenopus laevis* [13]. A comparison of several aspects of the composition of female germ-line cysts, such as its spatial organization, mode of interconnections between cyst components, participation of the cytoskeleton in cyst stability and oogenesis between *T*. *tubifex* and the above-mentioned species are presented in Table 1.

When we compare the multicellular germ-line cysts of *T*. *tubifex* with other non-annelid species, the similarity of the cyst architecture in the species studied here and in *C*. *elegans* is conspicuous (Table 1). The distal arm and loop of the U-shaped gonad in *C*. *elegans* is also composed of a single syncytial cyst. In both cases, the cysts are evidently polarized given that the syncytium contains cells that are in various stages of oogenesis at the same time. In *C*. *elegans* a common cytoplasmic core (gonad core) occurs in the center of the cyst, runs through the syncytial part of the gonad and each germ cell is connected to it by one intercellular bridge (rachis bridge) [22, 23, 43–47]. Similarly, as a rule, each germ cell in *T*. *tubifex* is also connected to a common cytoplasm by only one stable intercellular bridge. However, in contrast to *T*. *tubifex*, the rachis in *C*. *elegans* is cylinder-like and unbranched. Hirsh and colleagues (1976) stated that about 1,300 mitotic nuclei and 10–14 growing oocytes occur at the same time in the gonad of *C*. *elegans*. We found 2,000 cells per syncytial cyst on average in *T*. *tubifex*. The average number of oocytes that grow at the same time was eight. As was mentioned, two morphologically distinct germ cell categories regarded as nurse cells and oocytes have been described in *T*. *tubifex* ovaries. There are no morphologically distinguishable nurse cells in *C*. *elegans*; however, many experimental data suggest ovary meroism. Wolke et al. (2007) showed cytoplasmic streaming within the cyst in the *C*. *elegans* gonad. During this process, cytoplasmic materials flow from a region in the distal gonad where transcriptionally active pachytene-stage germ cells are localized into the proximal region towards the growing, transcriptionally inactive oocytes. Thus, the pachytene-stage cells can be considered to be a transient nurse cell population [47]. Additionally, it has been demonstrated that about half of the *C*. *elegans* germ line cells die in late pachytene [44], and therefore if these cells supply other germ cells via the gonad core, they function as “true” nurse cells [47].

The germ-line cysts that were found in *T*. *tubifex* show several striking analogous similarities with the syncytial cysts that are found in the telotrophic meroistic ovarioles of Hemiptera. However, the germ-line cysts in Hemiptera develop in a significantly different way than those in *T*. *tubifex* i.e. the cysts in hemipterans are branched with a prominent fusome [5, 48, 49], whereas in the developing cysts of clitellate annelids neither branchings nor a fusome were found [50]. In hemipterans, only one germ-line cyst exists in each ovariole (which is in our point of view, functionally equivalent to the *T*. *tubifex* ovary). There are fewer of germ cysts per syncytium in hemipterans (Table 1; e.g. up to 320 in *Psylla alni* (Psyllinea)) than in the sludge worm [5], although the germ cells are morphologically diversified into nurse cells and oocytes in both cases [27, 48, 51, 52]. The nurse cells in hemipterans occupy the apical zone of the ovarioles (the so-called tropharium), and linearly arranged oocytes usually grow below it (the so-called vitellarium) [5, 48, 49]. Interestingly, the central part of the tropharium is occupied by a large anuclear cytoplasmic area (the trophic core) and each germ cell in a cyst is connected to it. The trophic core is usually filled with bundles of microtubules [48, 51, 52]. Numerous nurse cells in the syncytial cysts in *T*. *tubifex* are equivalent to the hemipteran tropharia, whereas the cytophore is the functional equivalent of the trophic core.

### Cytoskeleton in syncytial cysts

The active roles of the microfilaments and microtubules during oogenesis have been experimentally demonstrated in different species. The cytoskeleton plays an integral role in oogenesis e. g.: in the maintenance of the structural integrity of all of the germ-line cysts; the transport of cell components (such as RNAs, macromolecules and cell organelles) from one germ cell to another through intercellular bridges (ring canals); the proper positioning of germ cell nuclei during this process (for references and details see Table 1). Two phases of this actin-dependent transport were distinguished in *D*. *melanogaster* – the first (slow) included the selective directional transfer through the ring canals into the oocyte, and the second (fast, called dumping or cytoplasmic streaming) in which the nurse cells transfer the remaining content (except for the nuclei) into the growing oocyte [7, 53–56]. Actin-dependent transfer has also been described in *C*. *elegans* oogenesis [47] (Table 1).

Our studies revealed that the extremely large syncytial cysts in the sludge worm are enriched with cytoskeletal elements such as F-actin and microtubules. Extensive actin bundles run within the cytophore and seem to reach individual germ cells. These bundles appear to form a scaffold and may play a structural role in the maintenance of a cyst’s integrity. Future experimental studies that are devoted to the transport of cellular components in *T*. *tubifex* using, e.g. injected particle tracking in living cells or toxins that alter the cell cytoskeleton will enable us to find out whether the prominent actin and microtubular cytoskeleton also plays an active role during oogenesis.

As in other germ-line clusters that have been studied [3, 57] the germ cells in *T*. *tubifex* are interconnected by stable intercellular bridges (ring canals) [27, this study]. Our results in the present study reveal that the ring canals in *T*. *tubifex* contain F-actin. Filamentous actin seems to be a widespread and common component of the ring canals that stabilizes the ring canals after their formation [3, 57, 58]. However, in some cases, such as in the fully developed male germ cysts of *D*. *melanogaster*, there is no F-actin in the ring canals [59]. Filamentous actin in intercellular bridges has also been recorded in other clitellate annelids such as branchiobdellid *Branchiobdella parasitica* [30] and the fish leech *Piscicola geometra* [34] and in several arthropod taxa, including Diptera, Hemiptera, Coleoptera and Branchiopoda [48, 57, 58, 60–62].

## Conclusions

The huge, multicellular germ-line cysts that were found in *T*. *tubifex* constitute a suitable foundation for future investigations, which will provide new data on the structure and functioning of germ-line cysts that have a central anuclear cytoplasmic mass. Numerous germ cells that diversify into oocytes and nurse cells morphologically, the extensive cytophore that is rich in cytoskeletal elements and the fact that this species can easily be reared in the laboratory also support the choice of *T*. *tubifex* as a good model for the analysis of germ-line cyst function.
